# Atlanta Society of Medicine

**Published:** 1888-04

**Authors:** 


### Society Reports.

### ATLANTA SOCIETY OF MEDICINE.

STATED MEETING, March 6, 1888.

The President, Dr. Virgil O. Hardon, in the chair.

Dr. GASTON reported two cases:

One being that of a young man whose chest was crushed by the front wheel of a loaded wagon, while the rear wheel passed over his abdomen; the other of a man whose shoulder was caught under a street-car wheel, causing fracture of the scapula.

The former was seen with Drs. Gerdine and Von Douhoff, in Athens, who gave him an account of intense suffering, profound shock and great difficulty of breathing; subsequently marked fever and pain in region of liver.

Dr. Von Douhoff made a puncture with an aspirating needle in this region and drew off an ounce of blood, but there was no evidence of laceration of the liver, nor even of contusion of its structure.

Patient was first seen by Dr. Gaston thirty-six hours after the accident, and in spite of the morphine which had been given there was still soreness over right thoracic region and tenderness on palpation over the liver. Pulse, 120; temperature, 102° F.; respiration 25 at 10 P. M. February 19th.

February 20th, 2:30 A. M.—Pulse, 100; temperature, 99½° F.; respiration, 22.

At 8 o'clock A. M. pulse, 80; temperature, 98°; respiration, 20.

At 4:30 P. M. pulse, 65; temperature, 98°; respiration, 20.

A fold of flannel wet with a stimulating liniment was kept constantly applied over chest and abdomen.

Bowels were obstinately constipated, but were finally moved after repeated injections of castor oil and warm water. Until February 22d his urine had to be drawn with a catheter; after that date he passed it naturally. The symptoms gradually subsided and the patient was entirely relieved.

It is evident that in compression of the body by wheels, the lungs and bowels are less likely to suffer than the liver, which by its solidity and fixity does not escape so readily; so that in this case the chief trouble seemed to be from the impression on this organ. There was some dullness over the right lung, but it did not develop into pneumonia.

The other case was seen in consultation with Dr. J. S. Bennett. It was ascertained while the patient was anæsthetized that there was a fracture across the inferior angle of the scapula, and another at the surgical neck just behind attachment of the acromion and coracoid processes. This allowed the shoulder to drop downward to a greater degree than is observed in fractures of the outer portion of the clavicle. According to Astley Cooper, fracture of the neck of the scapula means fracture through the *suprascapular notch*, a lesion which has unquestionably occurred, but the fracture above described is a very rare one, and is not described by any surgical authority; for this reason it has a special interest. There was considerable contusion of the soft parts, a punctured wound in the arm, and a lacerated wound in the hand, which Dr. Bennett had partially sutured. Indications for treatment are the same as for fracture of clavicle, viz., bandaging the arm to the side with a compress in axilla, the elbow being raised by a triangular sling. If the wedge under the arm is wide enough to extend beyond the axillary folds before and behind, there can be no pressure on the nerves and vessels in the axilla. This form of dressing was applied, together with compresses, over the scapula. It is expected that good, bony union, without deformity, will result in six weeks or two months. There is no indication that traumatic pneumonia or other consequences of a serious nature from the extensive contusions will occur.

Dr. NOBLE reported a case of extensive laceration of the cervix uteri. The interest in the case is centered on the peculiarity of the laceration. The patient came to him from Middle Georgia. The laceration was of eight years' standing, complicated with complete procidentia. The vaginal portion of the two lips (laceration bilateral), though reduced about fifty per cent., was more than an inch in extent from the vaginal junction. Above the vaginal junction the tear extended one and a quarter inches further on to the internal os, thus making a laceration of more than two and a quarter inches after a reduction of an inch in the everted lips. The tear extended from the cervical canal through the uterine substance into the broad ligaments on a parallel with the internal os, leaving, as it were, the peritoneum between her life and eternity. The vaginal rents healed up to the uterus, leaving a sharp, shoulder-like margin at the vaginal junction and a rectangular depression at the internal extremity of the wound. A small portion of the cervix was torn away and now remains attached to the side of the vagina completely isolated from the uterus. This piece was undergoing cystic degeneration.

[The President and other members stated that they had never seen such a laceration.]

Dr. ELKIN reported a case of subglenoid dislocation of the head of the left humerus, in which the deltoid muscle had been torn from its insertion on the shaft of the humerus. The patient was a man sixty-five years of age, who, on the 1st of last October, was knocked down while engaged in a fight, falling upon his hands and sustaining the above injury. The dislocation was easily reduced, and the limb, by means of a roller bandage, was fixed firmly to the side of the chest, the forearm being suspended in an ordinary handkerchief sling. At the expiration of two weeks the bandage was removed, and the patient instructed to use the arm gently until complete motion was restored. On removing the dressing he noticed at the insertion of the deltoid an indurated swelling about the size of a pigeon egg, which was more or less discolored, and elicited considerable pain when pressed upon or when the patient made an effort to raise the arm from the body, thereby bringing into play the action of this muscle.

Although the doctor could readily move the arm in any direction without pain to the man, yet the patient was unable to bring the injured limb from the body, and each attempt to do so was attended with much pain at the indurated spot. All the other muscles of the arm and shoulder acted perfectly, and seemed to have sustained no injury. Local applications in the form of liniments were rubbed over the joint and injured spot two or three times a day for sometime, but with no apparent benefit.

At the expiration of two months the action of the deltoid was unimproved, and there was still pain at its insertion when the patient endeavored to raise the arm.

On account of the disuse of the arm all the muscles became more or less atrophied, the shoulder lost its rotundity and the head of the humerus became quite prominent. On December 1st the doctor began the use of electricity. The galvanic current was applied to the injured limb three times a week for one month without any good result.

After this all treatment was abandoned, and the patient advised to use the arm gently as much as possible, and report the result. The case was seen three weeks afterwards, when the patient was able to raise the arm without pain almost at a right angle to the body. The indurated lump had disappeared and the arm was fast regaining its strength and usefulness.

Dr. COOPER reported a case of complete paralysis of the deltoid muscle following a dislocation of the right shoulder. The patient, a healthy young man, sustained a luxation of the right humerus in a railway accident in March, 1887; this was at once easily reduced by a physician who happened to be on the train, but, when the patient became able to use his arm again, he found that the power of abduction was totally lost. Pending a suit for damages against the road, his attorneys sent him to Dr. C. for treatment, and he came under his care in July, 1887. Examination revealed the fact that he was capable of executing every movement with his right arm, except the single one of raising the arm outward from the body. The right deltoid muscle was completely paralyzed, and would not respond to the strongest Faradic current the patient could bear. The muscle had wasted considerably and the skin covering it was cold. In this case the lesion was evidently an injury to the branch of the circumflex nerve which supplies motor power to the deltoid, either a complete laceration of the nerve, or else an extravasation of blood into its sheath, producing compression and consequent abolition of function. The treatment consisted in the application of electricity three times a week to the paralyzed muscle, in conjunction with the daily use of the hot and cold douche, shampooning and passive motion, the latter in order to prevent any ankylosis at the shoulder-joint. This treatment was rigidly persisted in for ten weeks without any improvement whatever occurring. The patient was seen again several months after treatment had ceased, but no amelioration could be noted. It is possible that the nerve fibres will yet be regenerated and the function of the deltoid muscle restored, but the outlook is not very encouraging.

Dr. Gaston said he had seen two cases in which the pressure of the dislocated humerus had obliterated the functions of the nerves of the arm, so that complete atrophy of the member in one case led to amputation for the removal of a useless appendage, and in the other case the arm remained permanently useless.

Dr. CHAPMAN reported a case of vicarious menstruation in a negro woman, aged 30. She has been married twelve years, but has borne only one child, a girl now nine years old, and to all appearance, healthy. He was called to see this woman during the early part of last January. At the time of his visit she was bleeding profusely from a syphilitic ulcer situated just above the internal malleolus of the right ankle. This ulcer was as large as the palm of the hand, and the escaping blood was of an arterial character. Upon inquiry he learned from the patient that the bleeding occurred only once a month and at the same time with menstrual discharge. This bleeding lasts for four or five days, and is so profuse that she uses bandages to stop the flow. The discharge from the uterus is slight, faintly colored and only occurs during the second or third day of the bleeding from the ulcer. She has had this ulcer for eight years, but the bleeding has occurred only during the last two ; before that the discharge from the uterus was normal. He has seen this patient at regular intervals since his first visit, and has found that the bleeding occurs only during the time of her monthly sickness. The veins of the leg are not in a varicose condition.

Dr. Hardon gave the history of a case of vicarious menstruation occurring in his practice, the hemorrhage taking place every month from the bronchial mucous membrane. There was no reason to suspect phthisis either from the examination of the chest or from the symptoms presented.

Dr. Noble stated that he had but little faith in most of the reported cases of vicarious menstruation. In quite a large proportion phthisis proved to be at the bottom of the trouble. He related a case corresponding to Dr. Hardon's, but stated that he found dullness at the apex of one lung, and that the patient improved under tonics; the cough subsided to some extent and the menstruation returned. He believes that undisputed cases of vicarious menstruation are rare.

Dr. Cooper detailed the symptoms in a case of vicarious menstruation in a healthy girl of eighteen. The hemorrhage occurred every month from the nasal mucous membrane. After two or three months of treatment she began to menstruate in the normal way, and the monthly nose-bleed ceased.

Dr. Hardon reported a case of labor presenting unusual complications:

Mrs. C. N., aged 35, white, married, pregnant for the fifth time, was seized with labor-pains May 26th, 1887. She had gone about three weeks beyond the time at which she expected to be confined. The pains continued with vigor and frequency through the night, the os slowly dilating and the bag of waters protruding, but the head failed to become engaged. I saw the case in consultation early on the morning of the 27th. I found the os dilated to the size of a silver dollar, the bag of waters protruding unbroken, the head resting on the brim of the pelvis, the face presenting with the mento-frontal diameter in the right oblique diameter of the pelvis. The cervix was soft and dilatable. I introduced my left hand into the vagina, and by making pressure upward upon the chin in the interval between the pains, I produced flexion of the head, so that the occiput presented in the L. O. A. position. With my hand in the vagina I held the head in this position until the uterine pains caused it to become engaged in the pelvis. During this manipulation the membranes were ruptured. The labor progressed slowly but steadily through the day, and I was again summoned to the case early the following morning. I found that the head had been born just before my arrival, but that the shoulders were arrested at the vulva, the right one resting upon the symphisis pubis and the left upon the perineum. With my finger in the axilla I brought down the posterior arm and by pressing the body of the child backward against the perineum liberated the anterior shoulder. The remainder of the body followed without any trouble. The child was an enormous one. The patient did well for three days, but at the end of that time developed a violent puerperal mania characterized by active violence towards her attendants and suicidal tendencies to such an extent that constant forcible restraint was necessary. I did not see her during this time. Three weeks after her confinement I was asked to take charge of her in the absence from the city of the attending physician. She had recovered from her mania, but had no recollection of anything since the commencement of her labor. All the intervening time was a complete blank. I found her in good condition in all respects, except that she complained of inability to retain her water. On examination I found that the urine escaped from the vagina, and careful inspection revealed a small opening in the vesico-vaginal septum about a half-inch above the neck of the bladder in the median line. The opening was barely large enough to admit a Simpson's sound. The edges had entirely healed, and there were no other cicatrices in the vagina. I thoroughly cauterized the edges of the fistula with nitrate of silver and directed the patient to use the copious vaginal douche of hot water four times a day. At the end of sixteen days, without other treatment, the fistula had entirely closed and the patient had normal control of her urine. A few days later she returned to her home in a neighboring city.

